# Changes in the pattern of service utilisation and health problems of women, men and various age groups following a destructive disaster: a matched cohort study with a pre-disaster assessment

**DOI:** 10.1186/1471-2296-9-48

**Published:** 2008-08-28

**Authors:** Rik JH Soeteman, C Joris Yzermans, Peter Spreeuwenberg, Toine ALM Lagro-Janssen, Wil JHM van den Bosch, Jouke van der Zee

**Affiliations:** 1 NIVEL, Netherlands Institute for Health Services Research, Utrecht, The Netherlands; 2 Department of Family Practice, University Medical Centre St Radboud, Nijmegen, The Netherlands

## Abstract

**Objectives:**

Female gender and young age are known risk factors for psychological morbidity after a disaster, but this conclusion is based on studies without a pre-disaster assessment. The aim of this study in family practice was to investigate if these supposed risk factors would still occur in a study design with a pre-disaster measurement.

**Methods:**

A matched cohort study with pre-disaster (one year) and post-disaster (five years) data. Community controls (N = 3164) were matched with affected residents (N = 3164) on gender, age and socioeconomic status. Main outcome measures were utilization rates measured by family practice attendances and psychological, musculoskeletal and digestive health problems as registered by the family practitioner using the International Classification of Primary Care (ICPC).

**Results:**

Affected residents of female and male gender and in five age groups all showed increases in utilization rates in the first post-disaster year and in psychological problems when compared to their pre-disaster baseline levels. The increases showed no statistically significant changes, however, between women and men and between all age groups.

**Conclusion:**

Gender and age did not appear to be disaster-related risk factors in this study in family practice with a pre-disaster base line assessment, a comparison group and using existing registries. Family practitioners should not focus specifically on these risk groups.

## Background

Disasters often have an effect on the victims' health and victims present more psychological and physical health problems as a result. Within this context, several risk groups may be distinguished, as gender and age, which have been described after many disasters [1].

Most of the studies found that women present with more health problems than men in the aftermath of a disaster related to earthquakes and hurricanes [2-7]. Some studies showed other results, however, in which men appeared to be more vulnerable than women [8,9].

In her review using 160 studies about the health problems after disasters, Norris [1] concludes that in 49 studies a statistically significant gender difference was observed in post disaster stress, distress or disorder. Of these, 46 studies found female survivors to be more adversely affected, especially for developing a Posttraumatic Stress Disorder (PTSD). In a meta-analysis Brewin found that when men and women were directly compared within the same study, women were more at risk of developing PTSD holding constant the type of trauma [10]. Finally, Tolin & Foa conducted a meta – analysis on sex differences in trauma and PTSD, using 290 studies published between 1980 and 2005. Their general conclusion was that females were more likely than males to meet criteria for PTSD, although females were less likely to experience potentially traumatic events [11].

Some studies on the effect of age in presenting post-disaster health problems showed that middle aged (40–65) victims were most distressed [6,9,12] and showed a higher utilization of health care services [13]. Two groups of different ages were compared in most of these studies and the results showed that the older group (65+ years) presented with fewer symptoms of distress or depression. The inoculation theory has to be mentioned in this context, viz. that victims with more experience of life and its major and minor (personal) disasters are more resilient to the effects of a "new" disaster than "inexperienced" victims [12,14-16]. Contradictory results are found too, however, and several studies have shown elderly Japanese, Polish and Australian victims of natural disasters to be more at risk of post-disaster distress than younger groups [17-19]. In general, however, older victim groups are more resilient to the effects of a disaster than younger groups [1].

Almost all studies referred to above are based on designs that did not use pre-disaster data and used a cross-sectional, retrospective design with short-term follow-up, using (self-report) questionnaires. In the reviews and meta – analysis mentioned above [1,10,11] it was suggested that the design of the study strongly influenced outcomes and results. Retrospective studies were associated with weaker effects for female gender and stronger effects for younger age and the effect size was greater when respondents were interviewed rather than given questionnaires. Epidemiological studies were associated with a significantly greater sex difference in PTSD than were convenience-sample studies.

Moreover, most studies discussed gender and age differences concerning PTSD, while in family practice (or primary care in general) this disorder is not often diagnosed. After disasters family practitioners often diagnose other psychological problems (anxiety, depression, disturbances of sleep, concentration or memory) and/or physical symptoms. In addition, we know of no studies in family practice of gender and age as possible risk factors for post-disaster health problems.

On 13 May 2000 a fireworks depot exploded in a residential area of Enschede, a city with 125,000 inhabitants in the eastern part of the Netherlands. As a result, 18 residents and 4 firemen were killed and about 1,000 people were injured. Some 1,200 victims lost their homes and personal belongings and had to be relocated for some years. Baseline data were available after the disaster, because the health problems of (future) victims and controls had already been registered by the family practitioner in the period prior to the disaster. This enabled us to investigate health problems longitudinally, therefore, with the inclusion of pre-disaster utilization rate and morbidity.

The aim of this study was to explore whether the supposed risk factors of female gender and younger age would (also) appear in a study in family practice in which a pre-disaster baseline measurement was available with a longitudinal design, without recall bias and using a comparison group.

We hypothesized that women and members of the younger age groups will have, for several years post disaster, elevated rates of psychological problems and physical symptoms and an increased utilization compared to their pre disaster baseline, to members of a comparison group and compared to men and older age groups.

## Methods

### Setting

Every citizen of the Netherlands is registered with one family practitioner (FP), who acts as a gatekeeper to secondary care. This means that patients affected by the disaster and their medical histories were already known to their FPs in the period prior to the disaster. All participating FPs were already using electronic medical records (EMR). Thus in this study, it was possible to collect data from one year prior to the disaster and the study period continued until 5 years after the disaster.

All 60 FPs in Enschede were asked to participate in this study and 44 of them agreed. The sixteen FPs who refused to participate gave three different reasons; six expected an increase in workload, nine had no victims in their practices, and one did not use an electronic data system.

Patients were informed about their FP's participation in this study by posters and leaflets in the waiting room and by announcements in the local newspapers. They were entitled to object to the use of their anonymized data, but nobody did. The study was carried out according to Dutch legislation on privacy. The privacy regulation of the study was approved by the Dutch Data Protection Authority [20]. According to Dutch legislation, neither obtaining informed consent, nor approval by a medical ethics committee was obligatory for this observational study.

### Matching variables

After the disaster (as after many others) it was problematic to identify exactly who had been directly affected by the disaster, not at least because of the various possible definitions of 'affected', including the concept 'exposed'.

To overcome this problem two external sources were used: persons were either marked as affected in the patient registration of their FP (using the zip-codes of the affected area or because being affected was mentioned in the patient – practitioner encounter), or were registered in the database of the municipal Information and Advice Centre (IAC); residents were for example registered here to acquire a new house and for financial compensation. The two databases were compared and inconsistencies were corrected. Despite our efforts, we are not completely sure that every single person in our study was directly exposed to the explosions, while we are pretty sure they were all affected. By way of precaution, we will not use 'victim', but 'affected resident'.

All victims had to be registered with one family practice during the entire study period, from 13 May 1999 until 13 May 2005 and 3168 affected residents were finally included. FP patients were included as member of a comparison group when they were not identified as affected resident (see above), so that we could relate our findings to normal fluctuations in utilization rate and morbidity over time. The comparison group were patients in the same practices involved in our study and they had to have been registered throughout the study period. They were matched with the affected residents on gender, age and health insurance, variables which were extracted from the FPs' electronic medical records (EMR). The type of health insurance was used as a proxy for socio-economic status (SES), because this is directly related to income in the Netherlands. Persons with public health insurance are presumed to belong to a low or medium SES category and they make up 64% of the general population [21]. Private health insurance indicates a high SES.

Groups of female and male affected residents were made and five age groups were constructed. The limits of the age groups were chosen on the basis of research in Dutch family practice [21,22]. Children younger than five years of age were not included.

### Dependent variables

The International Classification of Primary Care (ICPC), which is used in Dutch family practice, is compatible with the International Classification of Diseases (ICD-10) and with the Diagnostic and Statistical Manual of Mental Disorders (DSM-IIIR) [23]. ICPC is a multi-axial classification system in which it is possible to register problems and symptoms in the words of the patient ('the Reason for Encounter') as well as the diagnoses as objectivised by the family practitioner. Symptoms and diagnoses registered in the EMR during contacts with patients were extracted for this study every three months and were grouped in one psychological and two physical clusters (musculoskeletal and digestive) in accordance with the ICPC. The choice of these clusters was based on the results of other studies in this population demonstrating a relationship with the disaster [24-26]. The cluster of the psychological problems consisted of ICPC codes representing stress reactions, anxiety and depressive problems/disorders. The most prevalent ICPC codes within the pre – disaster psychological cluster represented depressive disorder, sleeping problems, anxious feelings and depressed feelings (constituting 64% of the cluster). By clustering problems and disorders specific information was lost, but we prevent coincidental differences between gender and age groups due to limited numbers. In the ICPC no specific code exists for PTSD. There is one code for all stress reactions, acute, transient as well as PTSD.

### Statistical analysis

The study period started one year before the disaster and lasted until five years post-disaster. Utilization of family practice care was calculated as the number of contacts (consultations, visits and telephone contacts) per patient – affected residents and members of the comparison group – in six one-year periods. A dummy variable was created with yes (= 1, at least one contact in a one-year period) and no (= 0, no contact in a one-year period). Morbidity of health problems in the three clusters was calculated as the number of affected residents attending their FPs with those problems.

Differences and trends in average utilization rate and percentage of morbidity for each group (combinations of affected residents and members of the comparison group with gender or age categories) in different years were calculated and tested using a logistic multilevel model for repeated measures (using the MLwiN software) and the logistic estimation was performed with second order penalized quasi-likelihood (PQL) approach with unconstrained level 1 variance, which made it possible to control for the autocorrelation between measurements in individuals (modelling the full variance/covariance matrix between measurement occasions at person level). The person cluster in the practices was also controlled for, by using the FPs as a higher level in the model. Our research questions are specified as a linear contrast function that captures the relevant changes between post-disaster versus pre-disaster years within one group of affected residents, compared to the referenced group of affected residents. It was subsequently tested whether the difference between these internal group changes differed from zero.

Ethical approval: in accordance with the privacy protection procedures of the Dutch Data Protection Authority.

## Results

The groups of affected residents and matched comparisons both contained 3164 persons, 52% of which were men (table 1). There were more women in the youngest groups and in the oldest groups (5–14 and 65+).

**Table 1:** Numbers of male and female affected residents registered with a family practice in a period of one year pre-disaster and five years post-disaster.

Age group in years	Male	Female
5–14	150	160
15–24	202	176
25–44	624	532
45–64	508	394
65+	156	262

### Gender

#### Utilization rates

Utilization rate was monitored during one pre-disaster year and five post-disaster years. Female affected residents and comparisons already had a higher utilization than male affected residents and comparisons before the disaster occurred. Both female and male affected residents had a significant post-disaster increase (table 2) in the first year (P < .001) compared to pre-disaster. The second year again showed a statistically significant difference in both female (P < .001) and male affected residents (P < .01). When the increases in the utilization rates for female and male affected residents were tested in the first two years, a significant difference (P < .01) was found in the second year alone, which means that the increase in utilization rate remained significantly higher in female affected residents. The increase in the first year was similar for both sexes.

**Table 2:** Utilization rate by male and female affected residents and members of the comparison group as number of contacts with FPs per year, one year pre-disaster and five years post-disaster.

Utilization rate		Male		Female	
		affected	comparison	affected	comparison
Pre-disaster	Year 0	3,69	3,06	6,61	5,44
Post-disaster	Year 1	5,21***	3,25	8,51***	5,63
	Year 2	4,73**	3,34	8,38***§	6,06
	Year 3	4,81	4,13	8,60	6,98
	Year 4	4,48	4,16	8,17	7,30
	Year 5	4,53	4,12	7,88	6,59

** P < .01, year compared with year 0

*** P < .001, year compared with year 0

§ P < .01, women compared with men within one year and related to year 0

#### Psychological problems

Psychological problems were analyzed per gender during the same period. Female affected residents had higher levels of psychological problems than males during the overall study period, including the pre-disaster period (table 3), and both groups of affected residents showed a statistically significant increase in these problems (P < .001) in the first post-disaster year. The psychological problems decreased moderately after the first year post-disaster. The difference with the pre-disaster year remained significant until the fourth year for men and until the third year for women. When the differences between the increases for men and women were tested, however, they did not appear to be significant, which meant that the increased morbidity of psychological problems post-disaster was similar for men and women, given the existing pre-disaster differences.

**Table 3:** Psychological, musculoskeletal and digestive symptoms in percentages of male and female affected residents and members of the comparison group attending their FP at least once per year, one year pre-disaster (year 0) and five years post-disaster (years 1 through 5).

Psychological symptoms		Male		Female	
		affected	comparison	affected	comparison
Pre-disaster	year 0	12,9	10,9	19,1	14,9
Post-disaster	year 1	40,8***	11,5	55,1***	18,4
	year 2	24,6***	12,8	33,5***	16,9
	year 3	24,0***	13,7	33,4**	20,4
	year 4	19,0*	12,7	28,8	20,8
	year 5	16,9	13,2	24,3	17,3

Musculoskeletal symptoms		Male		Female	
		affected	comparison	affected	comparison

Pre-disaster	year 0	23,0	19,8	29,1	23,9
Post-disaster	year 1	25,4	19,8	31,1	24,2
	year 2	22,8	18,8	30,7	24,8
	year 3	22,2	19,6	31,3	24,4
	year 4	20,1	19,4	27,1	24,4
	year 5	20,7	17,7	28,5	23,6

Digestive symptoms		Male		Female	
		affected	comparison	affected	comparison

Pre-disaster	year 0	12,2	10,1	14,9	14,1
Post-disaster	year 1	12,9	9,4	18,1	14,3
	year 2	11,9	9,6	16,6	13,3
	year 3	12,9	10,4	16,8	14,6
	year 4	13,2	11,7	18,7	15,4
	year 5	11,5	11,3	16,6	14,7

* P < .05, year compared with year 0

** P < .01, year compared with year 0

*** P < .001, year compared with year 0

#### Physical symptoms

No statistically significant increases were found in male and female affected residents when changes in musculoskeletal and digestive symptoms were compared between the pre-disaster year and the five post-disaster years. Nor were any significant differences found between the changes in both sexes (table 3).

### Age

#### Utilization rates

Utilization rates in five post-disaster years were compared with the pre-disaster year. The tests were implemented for all affected residents in five age groups and related to the comparison group (table 4). All age groups demonstrated a statistically significant increase in the first post-disaster year (5–14 years P < .05, all other groups P < .001) and this increase persisted in the second year in some groups (25–44 years, P < .001 and 44–65 years, P < .01) and even in the third year (25–44 years, P < .05). These increases in each age group were compared with the adjoining older group and with the mean of all older groups, but no significant differences were found in the changes between the pre-disaster year and the post-disaster year in all age groups.

**Table 4:** Utilization rate by five age groups of affected residents and members of the comparison group as mean number of contacts with FPs per year, one year pre-disaster (year 0) and five years post-disaster (years 1 through 5).

Utilization rate		Age groups									
		Age 5–14		Age 15–24		Age 25–44		Age 45–64		Age 65+	
		A	C	A	C	A	C	A	C	A	C
Pre-disaster	Year 0	0,97	1,61	3,20	2,42	4,51	3,48	6,51	5,19	9,44	9,28
Post-disaster	Year 1	1,69*	1,58	4,79***	2,46	6,54***	3,36	8,19***	5,84	11,12***	9,66
	Year 2	1,48	1,77	3,73	2,78	6,20***	3,67	8,19**	5,98	10,80	9,99
	Year 3	1,42	2,06	3,91	3,22	5,86*	4,12	8,30	6,76	12,54	12,88
	Year 4	1,45	1,97	3,05	2,91	5,34	4,43	8,13	7,23	12,23	12,66
	Year 5	1,55	1,74	3,05	2,65	4,84	4,03	8,35	6,77	12,17	12,43

A Affected residents

C Comparison group

* P < .05, year compared with year 0

** P < .01, year compared with year 0

*** P < .001, year compared with year 0

#### Psychological problems

Psychological problems in the post-disaster years were compared with those in the pre-disaster year and a statistically significant increase in psychological problems was found in all five age groups in the first year (P < .001, table 5). These significant differences persisted in the adult groups and in the elderly in the second year (25–44 years (P < .001), 45–64 years (P < .001), 65+ (P < .01)) and in the third year (25–44 years (P < .001), 45–64 years (P < .001), 65+ < .05)). A statistically significant difference was finally found in the adult group of 25–44 years in the fifth year (P < .001). No significant differences were found between the pre/post increases in all age groups.

**Table 5:** Psychological morbidity in percentage of five age groups of affected residents and members of the comparison group visiting their FP at least once per year, one year pre-disaster (year 0) and five years post-disaster (year 1 through 5).

Psychological problems		Age groups									
		Age 5–14		Age 15–24		Age 25–44		Age 45–64		Age 65+	
		A	C	A	C	A	C	A	C	A	C
Pre-disaster	Year 0	6,2	7,1	12,2	8,7	17,9	12,6	18,4	14,8	16,4	19,0
Post-disaster	Year 1	25,6***	5,2	41,2***	10,4	49,9***	15,6	53,9***	17,5	51,4***	20,4
	Year 2	14,0	7,4	22,2	11,2	31,9***	15,5	33,8***	17,3	28,0**	18,3
	Year 3	13,0	8,9	25,8	15,0	30,8**	16,3	31,0**	18,5	31,5*	25,0
	Year 4	12,4	7,4	19,4	12,7	25,9	17,0	26,3	18,8	25,2	23,4
	Year 5	8,4	6,1	18,1	10,9	22,5***	16,2	23,0	16,3	21,5	22,7

A Affected residents

C Comparison group

* P < .05, year compared with year 0

** P < .01, year compared with year 0

*** P < .001, year compared with year 0

#### Physical symptoms

No statistically significant differences were found in the first year post-disaster when the post-disaster musculoskeletal and digestive symptoms of five age groups were compared with their pre-disaster levels (see additional file 1). Again, no significant differences were found when all age groups were compared with their adjacent older age groups.

## Discussion

The aim of this study was to explore whether female affected residents were more vulnerable than male ones and whether younger age groups were more vulnerable than older groups to the effects of a man-made disaster in a longitudinal design with a pre-disaster measurement and a comparison group. Changes in service utilization and in morbidity as presented by patients in family practice were tested.

The main finding of the study is that no statistically significant differences were found between men and women and between various age-groups with regard to post-disaster increases in utilization rate, in psychological problems and in physical symptoms. We conclude, therefore, that as such, female gender and younger age were no risk factor in family practice following this disaster. The finding that women present a higher utilization than men in the second year alone was an unexpected one. It is hard to explain, because no gender differences in presenting with psychological problems were found in the same year.

This finding that female gender is not a risk factor after a disaster is in contrast with the findings of many other studies [1-7,27]. A difference between our study and previous studies on gender differences may be that the previous studies were often based on natural disasters with a sudden and fierce impact, e.g. earthquakes or hurricanes. Such disasters may cause more extensive destruction of housing and infrastructure than the man-made disaster in the present study and these large scale disasters may have an additional impact on women as breadwinners, having to raise children, or losing social support [11].

Some studies on gender, however, demonstrated results resembling those in our study. In a study on gender effects after 9/11 [28], a lifetime risk of post-traumatic stress disorder (PTSD) in women was found that showed that PTSD was not directly related to the attacks. In another study on 9/11, an excess burden of PTSD was attributed to female behavioural factors (e.g. acting as primary care-giver, experience of peri-event panic attacks) and biographical factors (e.g. previous unwanted sexual contact, recent history of mental problems) [29]. The disaster itself seemed to play a limited role in these studies. Another study concerning the effect of an air show disaster showed that gender did not act as a risk factor on post-traumatic stress symptoms [30]. These three studies were controlled for pre-disaster morbidity. One 9/11 study about female victims without a pre-disaster assessment found a relationship between social and economic circumstances and PTSD suggesting that women are not more vulnerable to PTSD than men [31].

After studying reviews and meta-analyses [1,10,11] we concluded that results of studies about gender being a risk factor for post-disaster utilization and morbidity (or not) were influenced by the study design. Retrospective studies were associated with weaker effects for female gender and the effect size was greater when respondents were interviewed rather than given questionnaires[10]. Epidemiological studies were associated with a significantly greater sex difference in PTSD than were convenience-sample studies [11]. Our design was not retrospective, no respondents were used (no 'recall bias') and epidemiological methods were applied. Based on the literature mentioned we hypothesized (strong) effects for women, although our study did not concern PTSD, but stress reactions, depressive feelings/disorders and anxiety feelings/disorders and physical symptoms. Moreover, the effect of demographic characteristics can not be thoroughly understood without controlling exposure and/or subjective appraisal characteristics. As mentioned before, privacy rules made it impossible to be 100% certain about the amount of exposure and subjective characteristics were not available because existing registries were used.

In our study, all five separate age groups presented post-disaster increases in psychological problems and utilization. These increases did not differ from one another, however, and so it appeared that all age groups were equally vulnerable to the effects of the disaster. This is in contrast with the finding of Norris in her review, which was that 88% of all studies of adult victims showed that younger adults were more adversely affected by disaster than older adults [1]. We found no results, therefore, to support the inoculation theory as presented in several studies showing a stronger resilience of elderly victims to the effects of a disaster [14-16]. In contrast to the present study, however, these studies were performed after natural disasters and two of them included high proportions of older adults [14,15]. High age elderly were compared with young age elderly, but these groups were pooled in one 65+ group in our study, because of the low numbers of victims in these groups. One of the flood studies was controlled for pre-disaster morbidity[15]. Age was studied in an adult group of victims in the study of an air show disaster referred to above, which was controlled for pre-disaster symptoms. Like gender, age did not appear to be a risk factor for post-disaster psychological problems in this study [30].

In summary, gender and (younger) age as such are not risk factors for presentation of post-disaster utilization or morbidity in the present study. Of the few studies that confirm our findings, two had a "pre-disaster" design similar to our study [15,30]. The studies that showed female gender and younger age to be risk factors were mostly based on large scale natural disasters and they did not perform pre-disaster assessments and or used a comparison group.

### Limitations and strengths

The present study has a strong design with pre-disaster data being used as a baseline measurement; as Norris stated in her review [1]: 'controlling for pre-disaster symptoms when assessing the effects of exposure yields the strongest design possible in this field of research'. As a consequence, we already had insight into pre-disaster health problems and the results of our study could be controlled for pre-disaster baseline values. Health data of affected residents and comparisons were also compared and a risk of recall bias was avoided as well by using FPs' electronic medical records instead of self-reported questionnaires.

Some issues relating to the present study need to be considered. Differences between affected residents and the comparison group already existed before the disaster occurred and affected residents presented more psychological and physical problems, in spite of matching with controls on socio-economic status, gender and age. Adverse health outcomes in the aftermath of disasters often originate in poor social circumstances that already existed before the disaster. In addition, disasters tend to happen in socially deprived areas with residents presenting more health problems or in areas that are particularly vulnerable to the effects of natural disasters [32,33]. On the other hand, the type of health insurance turned out to be an insufficient proxy for the socio-economic status of affected residents and members of the comparison group.

In this study, we did not have any information about whether the affected residents were directly exposed or not. We are aware that this is an opportunistic study which was limited by practical problems often encountered in disaster research. In this case, due to privacy regulations it was not possible to explore the 'individual exposure'. To overcome this problem two external sources were used: persons were either marked as affected in the patient registration of their FP (using the zip-codes of the affected area or because being affected was mentioned in the patient – practitioner encounter), or were registered in the database of the municipal Information and Advice Centre (IAC); residents were for example registered here for acquiring a new house and for financial compensation. Indirectly, there is evidence that affected residents were directly exposed to the disaster. After this disaster, besides surveillance in family practice, a survey was conducted using questionnaires. It was possible to combine the two databases (questionnaires and EMRs from family practice) for 994 affected residents (31.5% of the study group used here). On average, these persons reported 10.4 stressful experiences during the disaster (e.g. saw smoke, heard the explosions, saw the explosions, felt the shockwave, saw dead bodies) and analyses of SCL-90-R subscales and Rand-36 subscales showed that having encountered stressful experiences during the disaster was significantly associated with more problems on all subscales [34]. In another study on 649 affected residents (20% of our study group), 75% of them had high scores (>25) on the Impact of Event Scale [35]. These results were not confirmed in the comparison group. Finally, in a secondary analysis, it was found that prevalence rates of the comparison group resembled those of the general Dutch population, while the affected residents had higher rates on several health problems [36].

We may conclude that indirect evidence confirms that the labelling of the study groups reflects a distinction between individual exposure among the affected residents and no exposure among members of the comparison group.

Psychological problems were combined in one cluster, which might have resulted in loss of specific information. The choice of clustering patient's problems was decided in order to prevent coincidental differences due to the limited numbers of patients. On the other hand, symptoms of PTSD, anxiety disorder and major depression, which are all co-morbid with each other, were included in the cluster.

A risk of overrepresentation of post-disaster psychological problems could not be excluded. After all, the FPs in the study knew their patients and who was an affected resident and who was not. On the other hand, they knew whether a problem that was attributed by the affected resident to the disaster, was presented in reality before the disaster as well [32]. Moreover, recall bias could be avoided by the use of EMRs. Finally, the FPs were trained in the ICPC classification system and they received feedback on the quality of their registrations.

## Conclusion

In conclusion, the fireworks disaster appears to have dispersed its impact equally among male and female affected residents of all ages. In specific terms, neither women nor any particular age group were at increased risk of suffering the detrimental health effects of this man-made disaster in a residential area. In other studies concerning this specific disaster, it was found that having a pre-disaster history of psychological problems and disorders appeared to be the most important risk factor for post-disaster psychological as well as physical health problems [24-26]. In the first three years post-disaster being relocated due to the disaster appeared to be another strong indicator for disaster related health problems. Risk factors which appear in 'normal' primary care (gender, age, insurance type, ethnicity) did not have any extra effect of the disaster: post-disaster differences between these groups may be explained by pre-disaster differences.

After disasters family practitioners do not have to focus specifically on gender or on any age group post-disaster, but especially on those with psychological problems before the disaster and patients who lost their houses and personal belongings. As Freedy mentioned [37], after disaster 'family practitioners are key agents for providing information, remaining empathic, encouraging patients to seek and accept assistance (...) and repeatedly checking on disaster victims for up to (at least) 12 months'

Our study is one of the first which used a pre-post design and a longitudinal control-comparison design, using existing registries in family practice. It is important that this alternative design will be implemented after another disaster, collecting exposure data as well.

## Competing interests

The authors declare that they have no competing interests.

## Authors' contributions

RJHS originated and wrote the article, and assisted with the analysis. CJY supervised all aspects of the study. PS completed the analysis. TALML-J, WJHMvdB and JvdZ originated the study and provided feedback and suggestions throughout. All authors helped to develop ideas, interpret findings and review drafts of the manuscript.

## Pre-publication history

The pre-publication history for this paper can be accessed here:



## Supplementary Material

Additional file 1 Additional file 1. Musculoskeletal and digestive symptoms in percentage of five age groups of affected residents and members of the comparison group visiting their FP at least once per year, one year pre-disaster (year 0) and five years post-disaster (year 1 through 5). Click here for file
