# Supplementary material for: Changes in the pattern of service utilisation and health problems of women, men and various age groups following a destructive disaster: a matched cohort study with a pre-disaster assessment
Source: BMC Fam Pract. 2008 Aug 28;9:48. doi: 10.1186/1471-2296-9-48 (PMC2553410; doi:10.1186/1471-2296-9-48)
Supplement: Additional file 1 — Musculoskeletal and digestive symptoms in percentage of five age groups of affected residents and members of the comparison group visiting their FP at least once per year, one year pre-disaster (year 0) and five years post-disaster (year 1 through 5). [file 1471-2296-9-48-S1.doc]

**Additional file 1: Musculoskeletal and digestive symptoms in percentage of five age groups of affected residents and members of the comparison group visiting their FP at least once per year, one year pre-disaster (year 0) and five years post-disaster (year 1 through 5).**

| **Musculoskeletal symptoms** | | **Age groups** | | | | | | | | | |
| --- | --- | --- | --- | --- | --- | --- | --- | --- | --- | --- | --- |
|  |  | Age 5-14 | | Age 15-24 | | Age 25-44 | | Age 45-64 | | Age 65+ | |
|  |  | A | C | A | C | A | C | A | C | A | C |
| Pre-dis | Year 0 | 7,9 | 7,2 | 23,4 | 17,8 | 26,6 | 21,2 | 30,2 | 28,7 | 31,3 | 25,2 |
| Post-dis | Year 1 | 12,3 | 10,9 | 26,0 | 16,8 | 28,1 | 23,1 | 34,0 | 26,6 | 29,9 | 24,1 |
|  | Year 2 | 11,0 | 10,6 | 19,2 | 20,8 | 29,1 | 21,5 | 31,2 | 24,9 | 29,0 | 26,6 |
|  | Year 3 | 12,6 | 10,6 | 21,6 | 21,1 | 26,8 | 21,7 | 30,2 | 24,8 | 33,5 | 27,9 |
|  | Year 4 | 11,0 | 12,1 | 19,7 | 17,3 | 25,3 | 23,2 | 25,5 | 25,1 | 27,4 | 24,5 |
|  | Year 5 | 11,3 | 13,6 | 18,1 | 17,1 | 25,1 | 20,7 | 29,7 | 24,1 | 27,4 | 22,7 |
|  | |  | | | | | | | | | |
| **Digestive symptoms** | |  | | | | | | | | | |
|  |  | Age 5-14 | | Age 15-24 | | Age 25-44 | | Age 45-64 | | Age 65+ | |
|  |  | A | C | A | C | A | C | A | C | A | C |
| Pre-dis | Year 0 | 4,2 | 7,6 | 11,7 | 9,3 | 14,2 | 12,6 | 14,5 | 12,3 | 19,4 | 17,3 |
| Post-dis | Year 1 | 7,0 | 5,8 | 13,4 | 7,9 | 17,0 | 11,2 | 16,1 | 14,2 | 19,6 | 17,7 |
|  | Year 2 | 6,1 | 9,0 | 12,9 | 7,2 | 14,2 | 10,5 | 16,3 | 12,0 | 18,1 | 19,3 |
|  | Year 3 | 7,5 | 7,3 | 10,8 | 9,0 | 15,3 | 11,3 | 15,8 | 13,6 | 21,8 | 21,4 |
|  | Year 4 | 8,4 | 7,3 | 12,0 | 10,7 | 15,2 | 13,0 | 17,8 | 14,8 | 24,2 | 21,2 |
|  | Year 5 | 7,5 | 9,0 | 12,0 | 14,0 | 12,6 | 10,4 | 16,8 | 15,0 | 19,8 | 19,7 |

A Affected residents

C Comparison group
